# 3D Reconstruction of cellular images from microfabricated imagers using fully-adaptive deep neural networks

**DOI:** 10.1038/s41598-022-10886-6

**Published:** 2022-05-04

**Authors:** Hossein Najafiaghdam, Rozhan Rabbani, Asmaysinh Gharia, Efthymios P. Papageorgiou, Mekhail Anwar

**Affiliations:** 1 Department of Electrical Engineering and Computer Sciences, University of California, Berkeley, CA 94720 USA; 2 Department of Radiation Oncology, University of California, San Francisco, CA 94158 USA

**Keywords:** Biotechnology, Computational biology and bioinformatics, Oncology

## Abstract

Millimeter-scale multi-cellular level imagers enable various applications, ranging from intraoperative surgical navigation to implantable sensors. However, the tradeoffs for miniaturization compromise resolution, making extracting 3D cell locations challenging—critical for tumor margin assessment and therapy monitoring. This work presents three machine-learning-based modules that extract spatial information from single image acquisitions using custom-made millimeter-scale imagers. The neural networks were trained on synthetically-generated (using Perlin noise) cell images. The first network is a convolutional neural network estimating the depth of a single layer of cells, the second is a deblurring module correcting for the point spread function (PSF). The final module extracts spatial information from a single image acquisition of a 3D specimen and reconstructs cross-sections, by providing a layered “map” of cell locations. The maximum depth error of the first module is 100 µm, with 87% test accuracy. The second module’s PSF correction achieves a least-square-error of only 4%. The third module generates a binary “cell” or “no cell” per-pixel labeling with an accuracy ranging from 89% to 85%. This work demonstrates the synergy between ultra-small silicon-based imagers that enable in vivo imaging but face a trade-off in spatial resolution, and the processing power of neural networks to achieve enhancements beyond conventional linear optimization techniques.

## Introduction

Visualizing the 3D location of fluorescently labeled tumor cells in vivo is critical for intraoperative navigation to identify tumors beneath the tissue surface and harbored in deeper sites such as lymph nodes. Conventionally, this process is done post-operation, in a laboratory setting by using targeted fluorescent probes and markers to identify any disease from the tumor bed sample. This process is extremely time-consuming, and requires several days to return results, potentially putting the outcome of the treatment at risk. Recent engineering advances have resulted in several novel imaging platforms that allow this process intraoperatively and concurrent with the surgery^1–3^. However, these instruments are significantly cumbersome and not practical with today’s minimally invasive surgical procedures, especially in complex and hard-to-access tumor cavities. While these large instruments rely on sizeable optics and lenses for their high resolution and reliability, they can’t be miniaturized and made practical for surgical settings as a result of the inevitably rigid optical equipment. In addition, such large instruments naturally do not allow for assessment of treatment response, where cell migration into tissues is important to be monitored in real-time and outside surgical settings—another critical application. Therefore, a much less complex imaging platform with a smaller form factor is preferred. Recent advances in microscopy such as light-field microscopy^4–8^ have enabled imaging of smaller features and imaging within the tissue. However, these methods require specialized optical equipment incompatible with a minimally invasive procedure.

**Figure 1:**
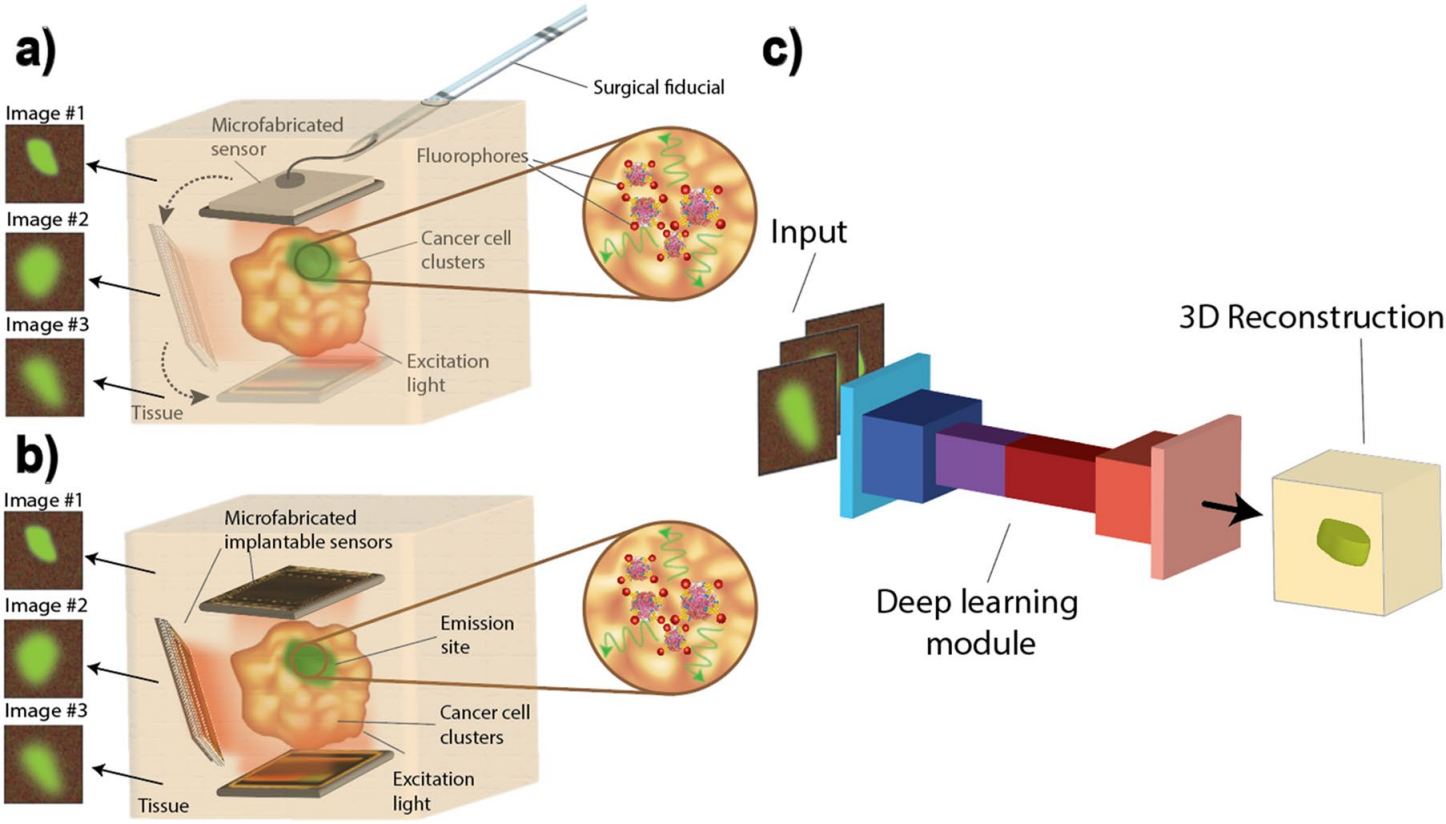
Concept of multiple visualization of the tumor using micro-imagers: (**a**) Multiple images taken by rotating the micro-imager intraoperatively. (**b**) Network of implantable micro-imagers to capture multiple fields of view. (**c**) Combination of neural networks and micro-imagers enabling 3D visualization and resolution enhancement.

Miniaturization of these platforms into electronic micro-imagers such as^9^ enables placement of imagers in hard-to-access regions, unlocking the ability not only to visualize microscopic disease intraoperatively in cavities up to several millimeters deep, but also to monitor cell dynamics and assess treatment in real-time and in vivo, with a network of wirelessly powered implants. Figures 1a and b illustrate how micro-imagers can provide a comprehensive visualization of the tumor of interest, with no disruption to the flow of operation or treatment for intraoperative and implantable applications such as in^10,11^, respectively. For intraoperative imaging, by rotating a surgical fiducial shown in Fig. 1a multiple image acquisitions from different angles of view can be obtained. Similarly, a network of implantable imagers can capture images from different angles of the target as shown in Fig. 1b.

While these ultra-small imagers are easy to integrate into surgical environments, unlike their larger counterparts, they lack a high enough resolution. Reducing the form-factor of the imager imposes a stringent limit on the size of the optical filters and focusing lenses being used, limiting their performance and the image resolution. Smaller imagers needed for in vivo use have often a higher background level (due to the lower rejection performance of smaller optical filters) and a lower focusing capability (due to the limited numerical aperture of their lenses). Safety limits also restrict the total photon budget allowed within the system, further constraining the capabilities of fluorescence microscopy in vivo. To obtain reliable 3D information using imaging instruments with these small form-factors, enhanced custom-made optical filters and lenses are required to replicate the same performance as their larger counterparts. These, however, are often difficult to manufacture, and image quality remains suboptimal relative to bench-top microscopes. Therefore, computational techniques that can enhance images from small form factor devices are in need.

Conventional image processing techniques involve variations of deconvolution, surface projection algorithms and noise enhancement methods, and the core of all these methods relies on a linear transformation of the image, that does not depend on anything other than the raw image data and the point spread function (PSF) of the imaging device and is procedurally blind to any prior knowledge of the specimen being observed.

The PSF, also known as the transfer function of the imaging system in spatial domain, describes the response of an imaging system to a point source of illumination^12^. In any linear image formation process such as fluorescence microscopy, the final image is a linear superposition of a series of point sources convolved with the PSF. Therefore, the original object can be retrieved by deconvolving the image with PSF of the imager.

One of the drawbacks of the deconvolution method is the calibration required for deriving parameters of the deconvolution function for images taken from each depth which limits image processing speed. This method poses difficulties for recovering images consisting of overlaid cell foci from different depths since every parameter requires optimization for a certain imaging depth. Moreover, the PSF, as a low pass transfer function, removes high frequency components of the original image, hence results in loss of sharpness, hindering generation of a fully recovered image after deconvolution. Even for cases where the image is mainly attenuated by the PSF response and still retains most of the high frequency components, applying inverse PSF amplifies high frequency noise degrading recovery of the original image. Much like deconvolution, every other linear image processing technique will suffer from similar issues.

To circumvent these limitations, a more agile approach is needed, namely a non-linear post-acquisition processing module that could incorporate the physiological and spatial information of similar tissue specimens within itself. The module can leverage this additional knowledge to restore the sharpness and resolution to the micro-imager’s suboptimal images, and provide insight into the 3D position of cells. Of all available architectures, deep learning modules can be by far the most adept at capturing physiological knowledge of cellular images.

Deep learning combines multiple layers of non-linear transformations, superposed with a complex yet structured network of coefficients to create powerful processing modules that can perform highly complex tasks, such as image enhancement, image classification and feature extraction. Yin et al.^13^ investigated characteristics of neuronal networks by extracting neuronal culture cluster information from microscopic images of neurons using machine learning models, and Chen et al. were successfully able to demonstrate label-free tumor cell classification using images of flow cytometry^14^. Deep learning allows breaking the tradeoffs of fluorescence microscopy and using the computational models to augment hardware complexity and improve upon optical limits, by using a large collection of training data to build the network^15^. Using adaptive network architectures such as residual neural networks (ResNets^16^) and convolutional neural networks (CNNs^17^), we present several applications for cancer imaging utilizing deep learning to enhance the resolution and capability of custom-made micro-imagers^18–22^. Figure 1c demonstrates how the symbiotic combination of neural networks and micro-imagers can not only restore the sharpness and resolution of the image, but also create a 3D visualization of the specimen at no additional hardware cost and create a highly reliable intraoperative imaging platform for margin assessment.

A key challenge to using a neural network in 3D cellular imaging of tissue is compiling a training dataset, since training deep neural networks requires access to a large set of training data from diseased tissue for each specific application. The impracticality of obtaining a large dataset, taken at varying depths, from tissue motivates synthesis of a diverse dataset of tumor cell images based on the morphology of real-life tissue samples, to leverage prior knowledge of the tumor cells. The synthesis method needs to be parameterizable to allow generation of an arbitrary large dataset by random selections of parameters that lead to images which are accurate representations of actual cells.

To address this, we first present a method to generate a large training set mimicking real-life specimens (a single layer of cells on slide), thus allowing our deep neural networks to be trained, as demonstrated in our prior work^23^. To replicate the 3D structure of the tumor, stacks of multiple layers of cells with 250 µm spacing within 1 mm from the sensor are generated. Since the lensless custom imager in^24,25^ is designed for contact imaging of tumor margins, 1 mm was set as the limit to demonstrate proof of concept. Next, we present 3 modules, tasked with identifying the depth of cell clusters -measured as the distance of the sample from the imager-, deblurring and enhancing the sharpness of the image, and finally, detecting cell presence within each layer of the specimen in 3D stacks. The last module incorporates a novel method of imaging using not one but two sensors viewing tissue from different angles to allow for three-dimensional imaging of the sample and providing insights into the spatial distribution of the cells in the sample.

## Materials and methods

### Dataset synthesis

To generate images identical to real-life cell foci, a coherent gradient noise generation method referred to as Perlin noise is used^26^. Perlin noise is a technique generally used to create natural appearing procedural textures, such as marble, wood, cloud textures for motion picture visual effects^27^.

To locate the tumor cell foci, a binary matrix is generated to represent a tumor mask with high values (> 0.5) indicative of tumor and low values (< 0.5) for non-cancerous background. Exploiting the natural structure of Perlin noise, a smooth cellular location map is achieved, populated with signal and background intensity values. Once the location of the cell foci is determined with the tumor mask, a tumor image is rendered by producing in-pixel signal and background intensity values based on mean and variance of real tumor images. A close correlation with real images is ascertained by making sure the statistical parameters stem from a representative range of parameters resulting from real data SNR calculations demonstrated in^23^.

Size of the tumor image is chosen to be 51 × 51, a form factor that parallels our lensless chip-scale CMOS imager for in vivo intraoperative imaging of cancer^9^.

### Modules implemented

A. Depth Estimator Module
  Nonlinear networks for depth estimation are often used in non-medical contexts, for traffic, navigation and security purposes^28–31^, and have proven to be quite capable of identifying spatial features and depths if properly trained prior to testing. To this end, we have built a convolutional neural network combined with a fully connected (FC) network to estimate the depth of images that emulate the custom-made imager’s resolution and PSF at various depths, randomly generated from 50 µm to 1.95 mm, with 100 µm increments.
B. Deblurring Module
  Obtaining high resolution images while maintaining a small size for the imager requires information beyond the mere raw data of the image, and an advanced knowledge of image contents. Knowing and having prior information about the underlying image data allows a more efficient recovery of the un-blurred image and insightful clinical information. A convolution/deconvolution neural network is able to absorb this information and embed it into the network using an appropriate training dataset.
  Here we created a 6-layer CNN trained to enhance and deblur corrupted images obtained with the custom-made imager. The training dataset used for this module was a compilation of single layers of emulated cell images to which the PSF of the custom-made imager was subsequently applied at different and randomly generated depths, ranging from 0 to 1 mm.
C. Cell Detector Module
  Building on the module for depth estimation described in (II.A), this module extends to localization of cells from different layers of the tissue and identification of their corresponding depth. The underlying advantage of multi-layer depth estimation exploiting machine learning models is distinguishing dim clusters of cells closer to the surface of the imager from bright cellular response far away from the device, a task hard to achieve without further processing. Multi-layer cell detection from a single 2D image with a precision better than 500 µm provides surgeons with high enough resolution to investigate the tumor bed.

Extracting multi-level depth information from the planar images of our lensless microscope on-chip eliminates the need for bulky optical lenses^32–34^. We present separate modules for detecting nonoverlapping and overlapping multilayer clusters of cancer cells. To generate the training dataset for nonoverlapping stacks of cells, pairs of emulated cell images from 2 different depth values are chosen randomly with a minimum difference of 500 µm from 0 to 1 mm. Initial cell images from each layer are convolved with the PSF of the custom-made imager and added together spatially to form a multilayer image. The overlapping regions are subtracted from the stacked image to ensure separation of the cells from each layer.

In addition to detection of cells in multilayer segregated clusters, 3D information from a more complicated structure with overlapping stacks of cells is explored in this manuscript. However, the accuracy of recovering 3D information using a single micro-imager is going to be very limited when extracting spatial information from farther layers. The decreasing optical signal and increased scattering and absorption and more importantly the PSF non-ideality will result in a very low signal-to-background ratio in the farther layers.

As a result, and to mitigate the high error rate of the model, we are proposing adding a second sensor to the imaging system, which is made possible by the ultra-small form factor of the sensor itself, allowing it to become fully implantable and therefore surgically practical. In our emulated experiments, the two sensors are placed 1 mm apart, on both sides of the emulated three-dimensional tissue.

Both the single and dual-sensor modules consist of a 6-layer CNN (3 convolution and 3 deconvolution layers) and their outputs are 4 binary input-sized layers depicting cell presence in each one, where [> 0.5] indicates cell presence and [< 0.5] indicates absence of cells. Due to the binary nature of the outputs, we can evaluate the accuracy of the outputs in terms of pixels incorrectly “labeled” (“existence” or “absence” of cells)- a metric we will later use to compare their performances.

For a reliable 3D imaging of tissue, it is necessary that the module maintains its sensitivity and specificity performance across the entire depth of the specimen, and as such, the limited performance of this module will preclude it from being used to acquire reliable 3D information and perform deep tissue imaging on samples that are more than a few hundred microns thick. In this work, sensitivity is defined by the ratio of pixels accurately predicting presence of cells over the total number of cancer cells in the ground truth images. Specificity, on the other hand, evaluates performance of the model in predicting absence of cells in the pixels indicated by the ground truth images to be empty of cells.

Amongst various applications of cellular level depth estimation, one of the more critical applications of microscopic imagers in oncology is to monitor and observe the movements and dynamics of the cells which represent the real-time response of tissues to therapy. The speed, direction, and features of the clusters of cells experiencing those dynamics are of significant clinical value, yet due to the complexity, cumbersomeness and optical limitations of intraoperative imagers, clinicians often have no choice but to do away with them. We present in our final module a 3D-reconstruction model architecture that is capable of capturing these dynamics. The model is used to evaluate the sensitivity to cell dynamics and movement across layers and verified quantitatively with test samples.

## Results

### Depth estimator module

The training dataset for this network has been compiled by initially generating raw and un-blurred images of a single layer of cells, after which the PSF of the microchip imager at different depths was applied onto them. The depths were randomly sampled from the range (0, 2 mm), with 100 µm step-size, thus allowing for 20 distinct depths.

**Figure 2:**
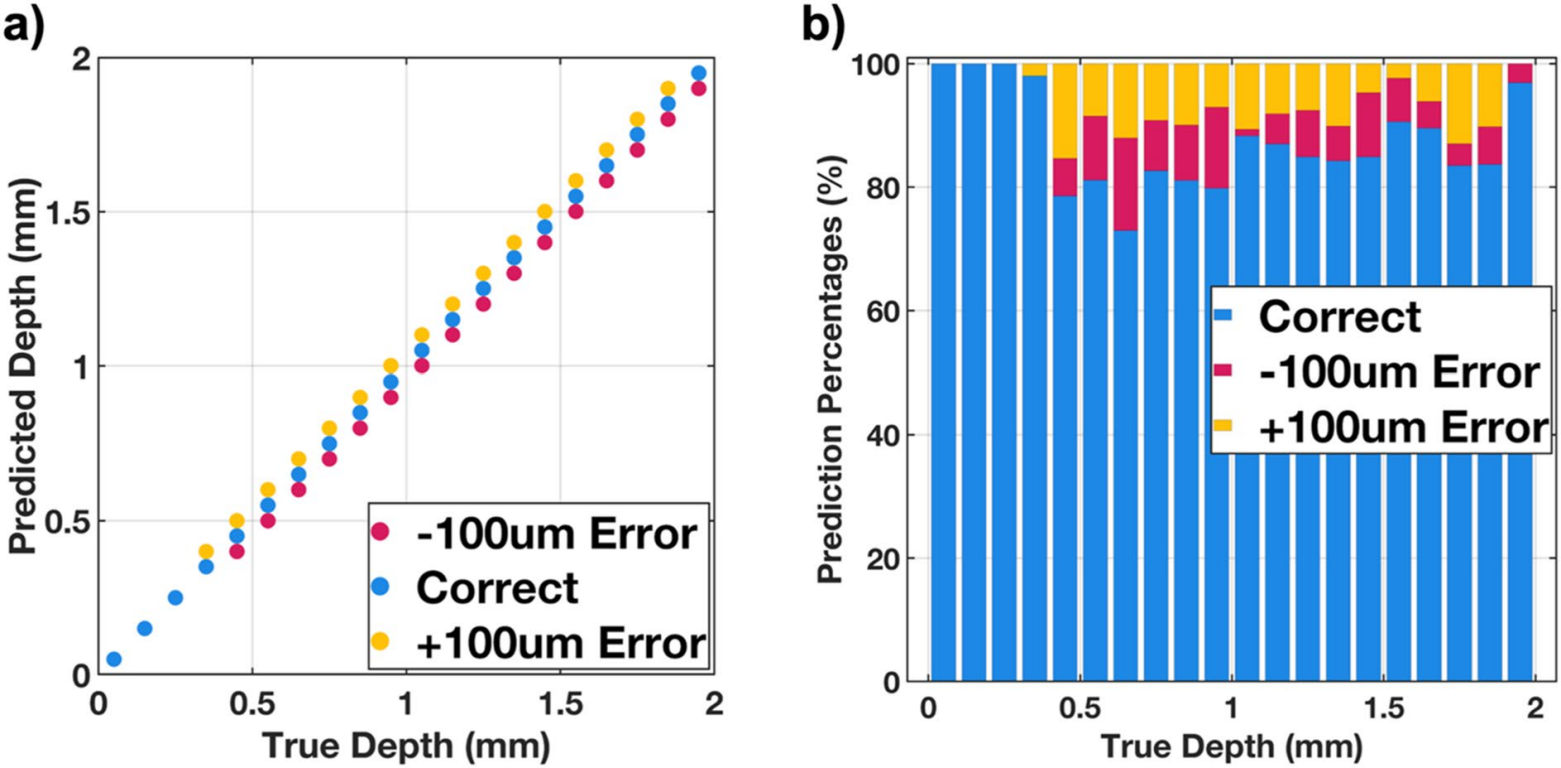
Performance of the depth estimator module: (**a**) Predicted values for every depth in the test dataset. (**b**) Test accuracy and loss for depths ranging from 50 µm to 1.95 mm with 100 µm step sizes.

After training, the module was tested on 1000 randomly sampled images at various depths ranging from 50 µm to 1.95 mm, and the prediction performance is shown in Fig. 2. Figure 2a shows the occurrence of all the predictions in the test dataset, and Fig. 2b illustrates the detailed statistical performance of it. The module was able to identify the depth of the test images with a maximum error of 100 µm, enough to enable resection of tumors, even below the tissue surface.

If the depth resolution is assumed to be 100 µm, then the accuracy rate of this module would be about 87%, with the accuracy growing closer to 100% as the specimen is closer and closer to the imager.

### Deblurring module

**Figure 3:**
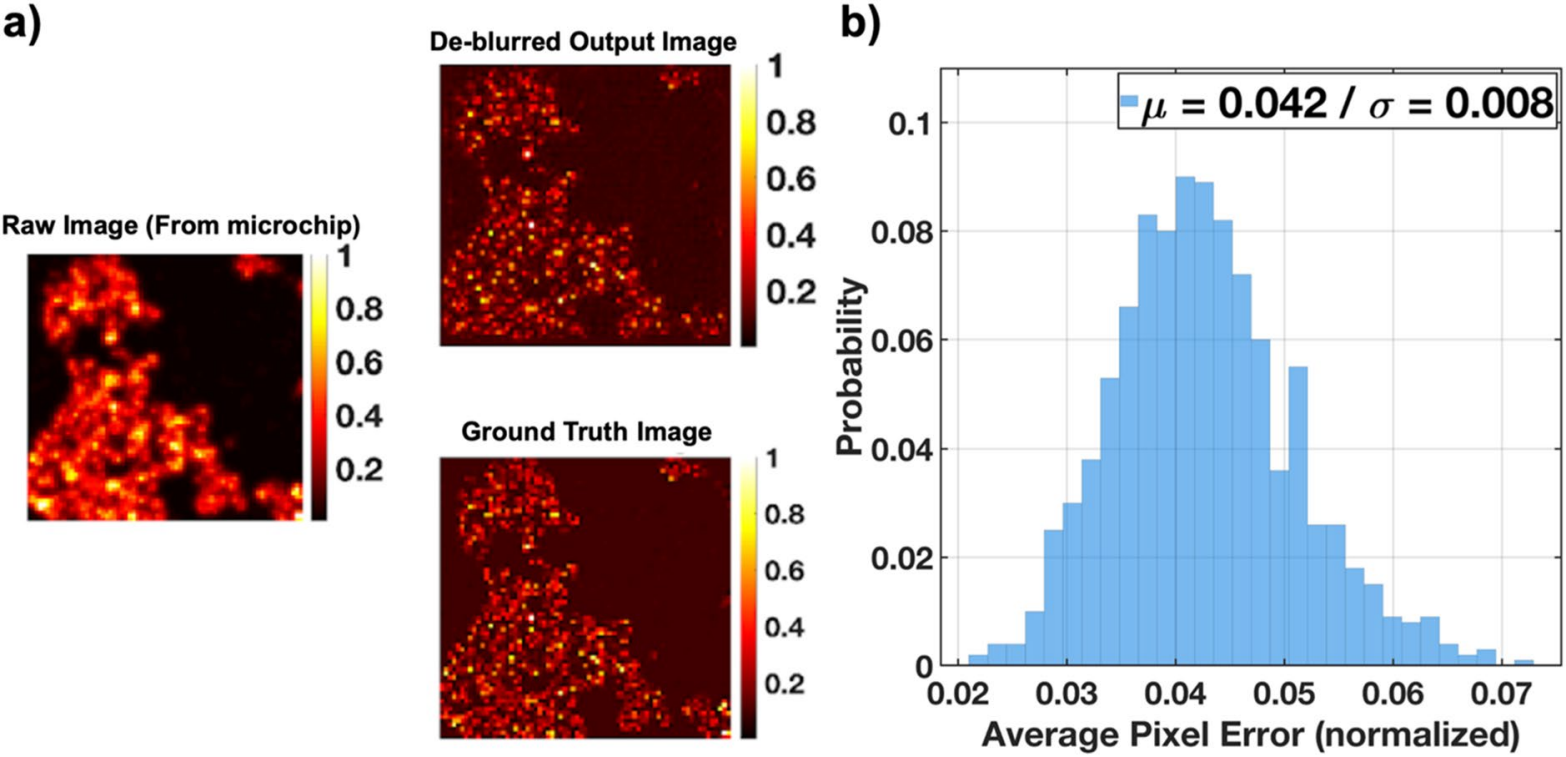
Performance of the deblurring module: (**a**) Network input and output images for a test sample in comparison with the corresponding ground truth image. (**b**) Distribution of average pixel error for 1000 test samples with a mean error of 4.2%.

We trained a 6-layer (3 convolution/3 deconvolution) network on a training dataset and tested the performance of the module on 1000 test samples and Fig. 3 illustrates the performance of the module. This module is able to extract the un-blurred image from a blurred input and restore sharpness and original resolution to the image, by relying on embedded prior image information and content-aware non-linear transformations. A sample test performance is shown in Fig. 3a.

The statistical accuracy of this module is illustrated in Fig. 3b which shows the average normalized deviation of pixels from their correct value is about 0.04, which translates to 4% estimation error.

### Cell detector module

**Figure 4:**
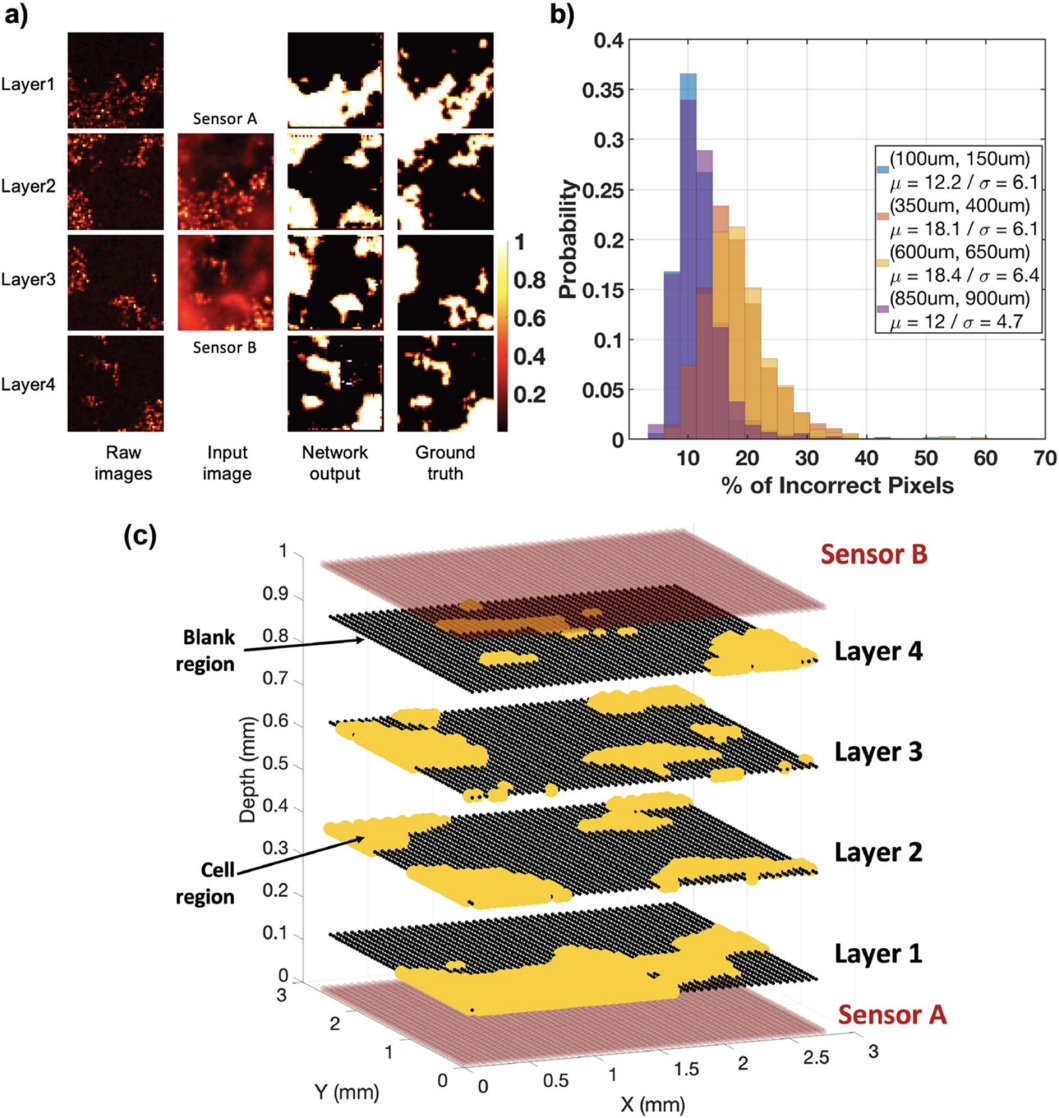
Evaluation of the CNN with two sensors: (**a**) Overlaid input images from 4 raw images corresponding to each layer before applying the PSF, network output images and ground truth depth maps. (**b**) Distribution of average pixel error for each layer with averaged error rates of 12.2%, 18.1%, 18.4% and 12% for layers 1 to 4, respectively. **(c)** Spatial (3D) reconstruction (using network outputs) of the sample test input, where black, yellow and red respectively represent blank (empty of cells) spaces, regions containing cells, and sensor locations.

**Figure 5:**
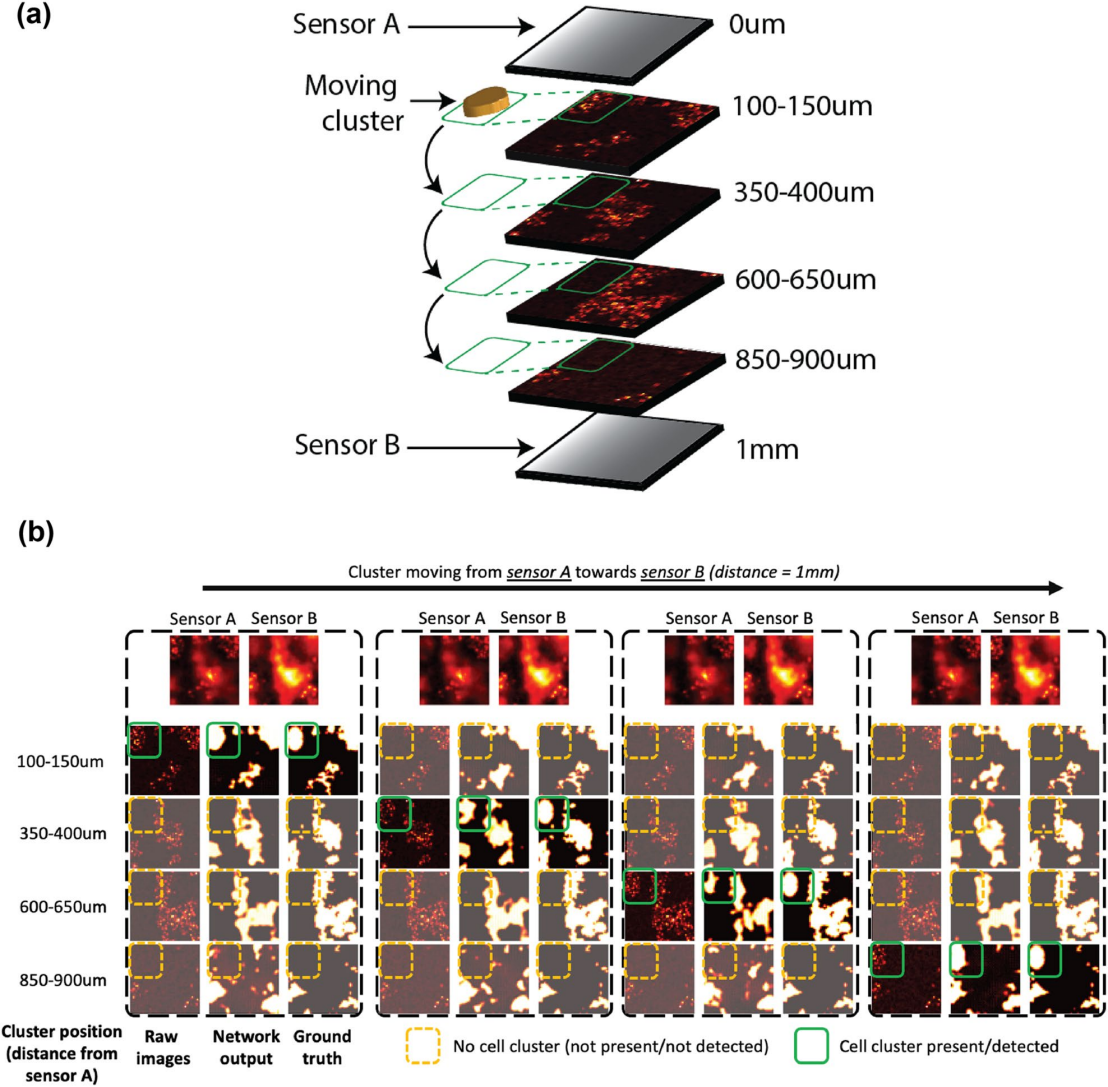
(**a**) Test setup for modeling dynamics of a moving cell cluster. (**b**) Outputs of the compounded module with the moving cell foci at each layer including the deblurred images at each depth, the two sensor images captured that serve as input to the module, the network outputs and the ground truth cell maps at each depth.

A. Non-overlapping Multi-layer Depth Estimation
  Similarly, a 6-layer (3 convolution/3 deconvolution) network is trained on the dataset and the performance is evaluated on 1000 test samples. Figure S1 shows the overlaid image from the stacks, the corresponding depth map and finally the depth map predicted by the model. Figure S1 depicts the error distribution of the depth map predicted by this network with an average normalized deviation of pixels from their correct value to be about 6% with a standard deviation of 10%. Having established a model that successfully identifies two-layer depth map of tumor cells within a normal tissue background, we aim to show that we can generalize 3D localization to a more comprehensive case discussed in the next section.
B. Overlapping Multi-layer Detection
  i. CNN with single sensor: The first multi-layer cell detection module is based on a single sensor, observing a stack of 4 layers of cells that are randomly spaced between 0 and 1 mm away from the sensor itself, where each layer is at least 200 µm away from the adjacent layer- to allow full coverage of the [0,1 mm] three-dimensional space with only 4 layers. Individual layers are uncorrelated, and have randomly generated intensities and background levels, allowing for a realistic emulation of tissue. Upon applying the corresponding PSF to each layer, the 4 images are summed and combined into one final image that constitutes the sensor’s output and will serve as raw input to the module.
    After training, the module evaluated over 1000 distinct test inputs, and the corresponding input and outputs for one sample image are shown in Fig. S2. The distribution of the performance is also shown in Fig. S2. Our performance metric reveals that the first layer, which is the closest to the sensor, has a lower error rate of 28%, and the performance degrades with farther layers, with the rate of inaccuracy remaining well above 37%.
    The distribution of the performance illustrated in Fig. S2 is shaped by the random overlap of cells in the four layers. A deeper analysis of the individual data confirmed that the cases with lower counts of incorrect pixels observed in the far layers (seen in Fig. S2) are caused by the subsequent cell layers overlapping significantly with the closest one, resulting in special cases and lower than usual error rates.
  ii. CNN with two sensors: The effect of adding the second imager, which is possible with the current form factors of these imagers, to the opposite side of the target under test is explored with the generalized 4-layer case and the improvement of accuracy is reported.
  iii. Similar to its single-sensor counterpart, and using the exact same network, we evaluated the module over 1000 distinct test inputs and the corresponding inputs and outputs for one sample image set is shown in Fig. 4a. The impact of adding a second sensor can be observed by comparing the outputs shown in Fig. S2 and Fig. 4a, quantified in Fig. 4b. Figure 4b illustrates the performance distribution of the 2-sensor module, and as expected, the first and last layers have very similar performances, as do the two middle ones, and this network can achieve a 12% error rate (in the two closest layers), which is less than half of the error rate of its single-sensor counterpart. Adding a second sensor reduces the error in the two middle layers – farthest from the sensor – reducing it from 40 to 18%.
    Upon extracting and identifying the regions in each layer that include cancer cells, we are then able to reconstruct a spatial representation of the stacked sample in space and provide a 3D visualization of the specimen being imaged. Illustrated in Fig. 4c, using the outputs shown in Fig. 4a, we have reconstructed the stacked sample, identifying the zones where cancer cells were detected in each layer. For a complete representation of the proposed imaging platform, the two sensors are also shown in Fig. 4c, separated by the 1 mm thick stack of cell layers in between.
  iv. ResNet + CNN with two sensors: Here we present a module that can not only identify cell clusters across all depths but is also very sensitive to small changes in the specimen, i.e., resulting from cluster movements. Using a more enhanced neural network, this module is able to identify cluster movements between layers and help visualize the dynamics within the specimen. In the previous section we introduced a two-sensor architecture to significantly improve the accuracy of the module. Here we preceded a 2-layer CNN network with a pre-trained 18-layer ResNet architecture^35^, and after training, evaluated the performance of the compounded network on the test dataset. The architecture of the network and its input images and output depth maps are shown in Fig. S3. The test dataset includes 100 distinct and randomly generated group images, in which a cluster of cells within the three-dimensional space moves across the different layers, going from sensor A towards sensor B, thus replicating a physiological dynamic of a real-life migrating cluster of cells (such as immune cells migrating into a tumor, or a metastatic deposit migrating or dividing within tissue). Figure 5a shows the diagram of the described scenario and how the cluster (and only the cluster) moves across the layers.

The performance distribution of this module is shown in Fig. S4 and it can be seen that the two closest layers have a very low error rate (~11%) while the two middle ones have slightly higher rates (~16%). However, both show noticeable improvements compared to the CNN-only network case.

**Figure 6:**
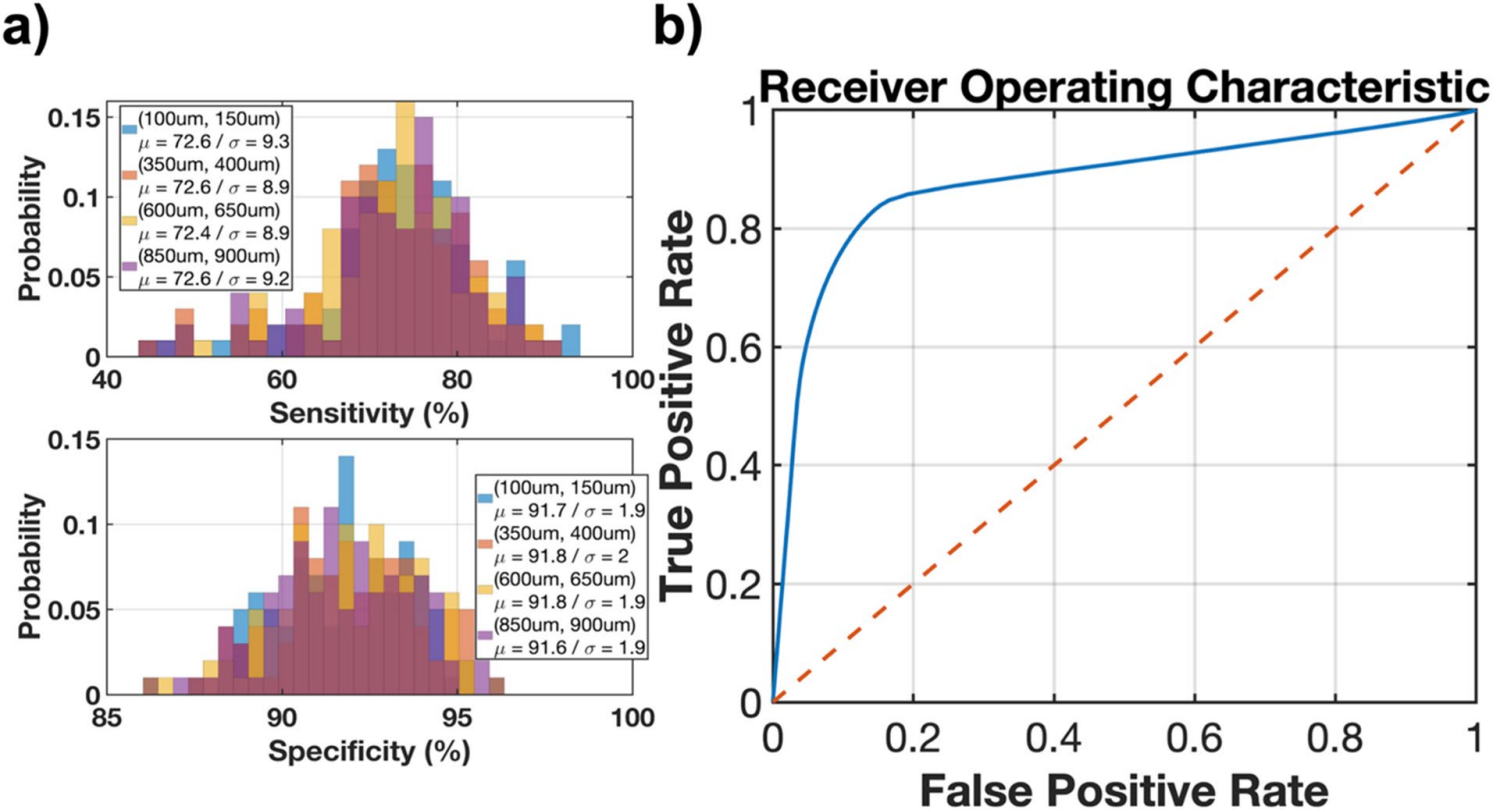
Performance of the compounded module with the moving cell foci: (**a**) Sensitivity and specificity of the ResNet + CNN with 2-sensor network in detecting dynamics of the moving cell cluster for each layer. (**b**) Receiver Operating Characteristic (ROC) of the model averaged for all layers.

The performance and outputs of the compounded module are shown in Fig. 5b. Figure 5b includes 4 sections, each one of which includes the raw (unblurred) images at different depths, the network outputs, the ground truth image and the two sensor images captured that serve as input to the module. The cluster of cells marked in Fig. 5b moves across the 4 layers (from left to right), altering the 2 sensor images every time a layer change occurs. The module tracks the cluster with an average sensitivity of 72.6% and specificity of 91.7% for all of the 4 layers as shown in Fig. 6a. The Receiver Operating Characteristic (ROC) for the average performance of the model across all layers is shown in Fig. 6b.

## Discussion

The modules presented in this work demonstrate the capabilities of neural networks in providing real-time enhanced image resolution and valuable 3D clinical information from currently available imaging systems and platforms. This project incorporates 3 modules featuring identification of the depth of a single image, deblurring and enhancement, and localizing cancer cell foci within each layer in both overlapping and non-overlapping stacks of cells. A novel method to synthesize a large representative dataset of real-life chip-scale images of the tumor is utilized for training deep neural networks. We presented a novel imaging platform by placing two sensors on both sides of a tissue stack to improve the error rate of our depth estimation algorithm compared to the case with only sensor. The performance of the three modules described in this work have been summarized in Table S1, illustrating that the addition of a second sensor to a 6-layer CNN significantly reduces the error rate in the middle layers -by up to 55%. In addition to that, we also trained a 2-layer CNN preceded by an 18-layer pretrained ResNet and the introduction of the ResNet network further reduced the average error rate from 15.2% to 13.5%. Table 1 summarizes the performance of the image enhancement and depth estimation techniques presented in this work in comparison with state-of-the-art supervised monocular depth estimation models for single images from NYU depth dataset^36^. Depth estimation for indoor environments eliminates the need for detecting stacks of overlapping objects^37–39^ while it is instrumental in detecting cell clusters in tissue samples. The cellular level depth estimation work presented in^40^ relies on multiple acquisitions from different depths of focus, challenging for intraoperative applications. This work achieved accuracies of 87% for single depth estimation for depths ranging from 0 to 2 mm, 95.8% for deblurring and resolution enhancement of images taken up to 1 mm away from the imager, 93.8% and 86.5% for cell localization and depth estimation of non-overlapping and overlapping stacks of multiple layers of cells within 1 mm of the device, respectively. To verify the performance of the model in dynamic applications, the network was also tested for identification of moving cell foci within multi-layer stacks of cells. The module was able to identify presence of the cluster with a sensitivity and specificity of 72.6% and 91.7%, respectively, achieving minimal “bleed-through” between adjacent layers. Having demonstrated the synergetic performance of the system containing our customized image sensor^9^ with the computation power of neural networks, for future work, a more comprehensive study is needed to optimize architecture of the neural network using structures such as encoder-decoder based CNNs^41^ or self-attention feedback networks^42^.

**Table 1:** Comparison of this work with current depth estimation models.

	This work				^37^	^38^	^39^	^40^
Application	Single-layer depth estimation	Deblurring	Multi-layer depth estimation		Depth estimation	Depth estimation	Depth estimation	Cell invasion depth estimation
			Non-overlapping	Overlapping				
Architecture	CNN	CNN	CNN	RES + CNN	RES + CNN	Multi-scale deep network	Encoder-decoder	Gradient convergence
Single acquisition	Yes	Yes	Yes	Yes	Yes	Yes	Yes	No
Dataset	Synthesized 2D images	Synthesized 2D images	Synthesized 2D images	Synthesized 2D images	NYU depth	NYU depth	NYU depth	3D cancer cell invasion assays
Average accuracy (%)	87	95.8	93.8	86.5	91.7	82.3	95.7	89.6

**Figure 7:**
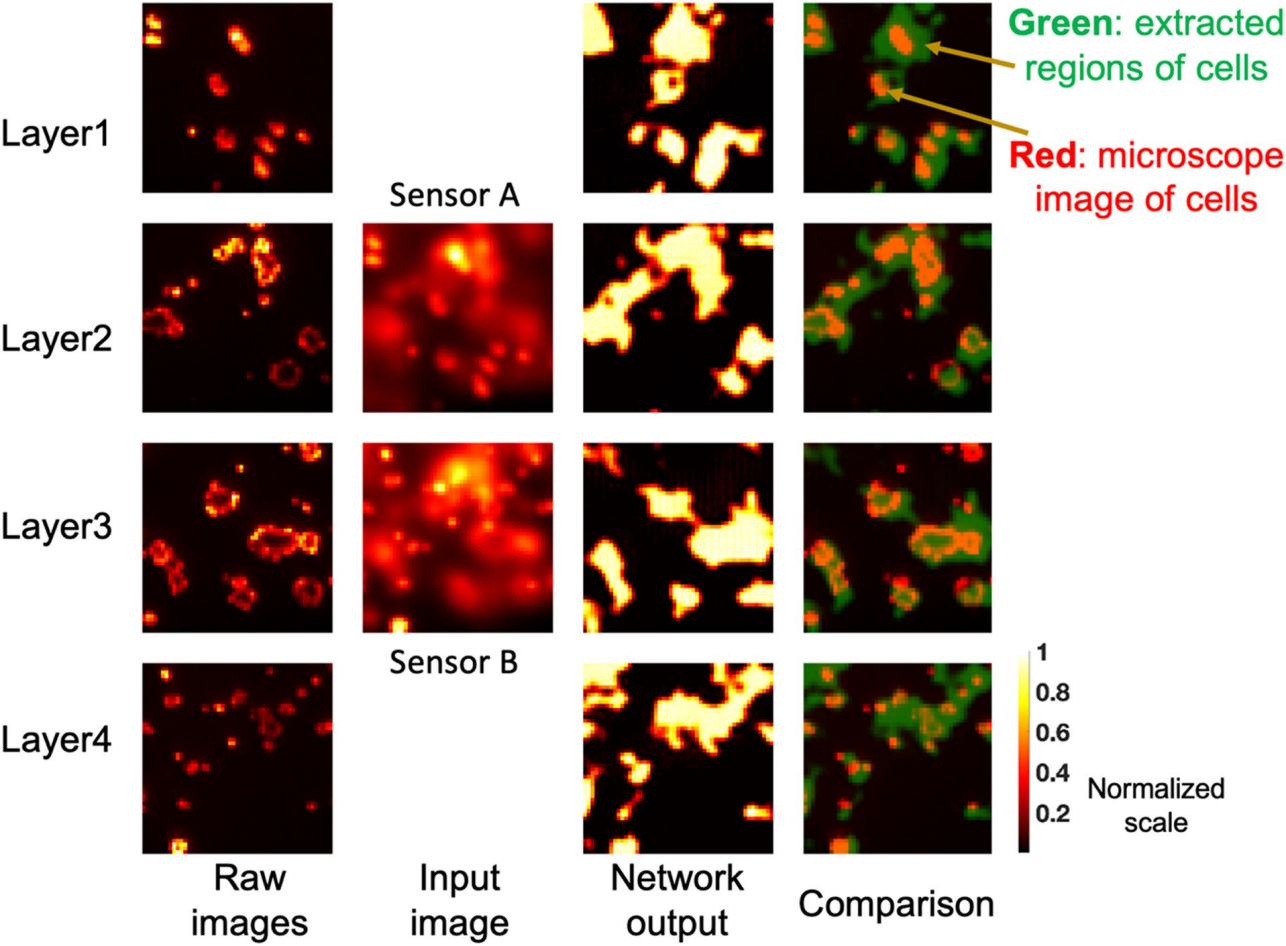
Evaluation of the CNN with two sensors on real-life cancer cell slides: (from leftmost to rightmost column) input images from 4 raw microscope images corresponding to each layer before applying the PSF, sensor images, network output images showing regions where cells were detected, and overlay composite image of network output and microscope images for each corresponding layer.

There are several limitations to our work. While we ensured our datasets replicated as close as possible to a real-life sample set, a synthesized dataset was used. However, our goal is to demonstrate proof of concept with this technique, and this approach can be repeated for any available cell imaging data set by retraining the neural networks on that dataset. Nonetheless, despite the synthetic dataset, the final model (ResNet + CNN) is still able to achieve a high level of performance when applied to real-life images, as seen in Fig. 7. While gathering a large size of real-life images of cancer cells is beyond the scope of this work, we have applied the module on a limited number of cancer cell slides. These specimens have first been imaged on high-resolution fluorescence microscope (shown in the first column in Fig. 7), after which each one has been assigned a randomly selected depth and was applied the corresponding PSF. After applying the PSF, all 4 layers were combined (added) to generate the sensor image. A similar procedure is also carried out for the second sensor. The sensor images obtained are shown in the second column of Fig. 7. The network output -illustrating regions where cells were detected in each layer- is shown in the third column, and an overlay composite image of the outputs with the microscope images is also shown in the rightmost column in Fig. 7, showing an almost perfect level of localized cell detection.

An additional limitation of this work involves partial modeling of optical path non-idealities. While the PSF accounts for the optical divergence of the light path, it does not take account for the scattering and absorption of the tissue medium. A further enhanced version of the module can be trained on a dataset that incorporates the effect and impact of scattering, absorption, motion deformation, non-uniformity of the laser beam and additive noise in the tissue as described in^43^. While an improvement, those effects can however largely be mitigated by using a longer wavelength process with the corresponding fluorophores, such as an ICG or IR-800 dye, as reported in^44–46^.

The superior representation capability of neural networks also allows for a far more complex network of sensors to be used than only two, and thus pushing the limits of detection and resolution even further than what has been presented in this work. Having readily available ultra-small sensors to be intraoperatively used, we can ultimately harness the power of machine learning in extracting valuable 3D information from a collection of easily obtained raw images.

## Conclusion

Obtaining high-resolution, cellular-level information from in vivo images of tissue is critical in oncological applications. To the best of our knowledge, this is the first work that incorporates deep neural networks for depth estimation from single cellular-level images. A large synthetic dataset representative of real cancer cell images enabled training of deep neural networks for single and multilayer depth estimation and image deblurring and resolution enhancement. Accuracies of 87% for single-layer depth estimation, 95.8% for deblurring, 93.8% and 86.5% for cell localization and depth estimation of non-overlapping and overlapping stacks of multiple layers of cells are achieved, respectively. The novel imaging platform presented here leveraged placing two sensors in tissue enabling high depth estimation accuracy for interoperative applications, which is made entirely possible by the ultra-small form factor of our custom-designed micro-chip sensor.

## Supplementary Information

Supplementary Information 1.

Supplementary Information 2.

## Data Availability

The dataset that was generated and used during this study is publicly available here https://github.com/rozhan-r/3D-Reconstruction-of-Cellular-Images.
