# Supplementary material for: 3D Reconstruction of cellular images from microfabricated imagers using fully-adaptive deep neural networks
Source: Sci Rep. 2022 May 4;12:7229. doi: 10.1038/s41598-022-10886-6 (PMC9068918; doi:10.1038/s41598-022-10886-6)
Supplement: Supplementary file 1 — Supplementary Information 1. [file 41598_2022_10886_MOESM1_ESM.docx]

# Electronic Supplementary Material

# **3D Reconstruction of Cellular Images from Microfabricated Imagers using Fully-Adaptive Deep Neural Networks**

Hossein Najafiaghdam^1^, Rozhan Rabbani^1^, Asmaysinh Gharia^1^, Efthymios P Papageorgiou^1^, Mekhail Anwar^2^

^1^Department of Electrical Engineering and Computer Sciences, University of California, Berkeley, CA 94720 USA (e-mail: hossein_najafi@berkeley.edu, +1-510-816-4455)

^2^Department of Radiation Oncology, University of California, San Francisco, CA 94158 USA

# **Supplementary Figures**


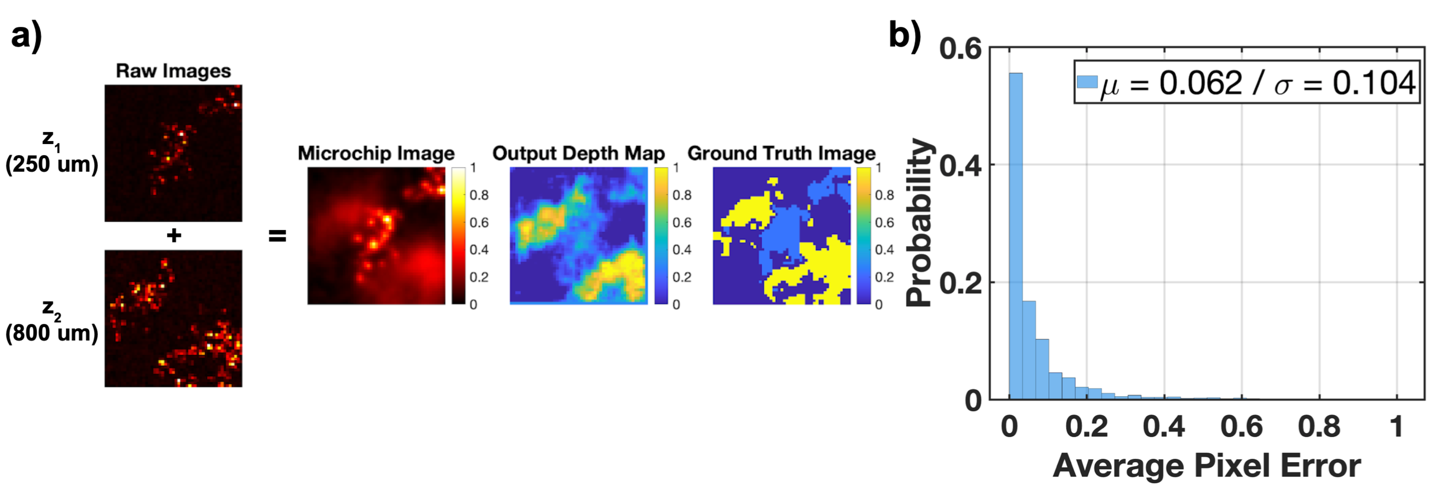


Figure S1. Performance of the cell detector module for non-overlapping stacks of cells: (**a**) Raw images at each depth before applying the PSF, network input and output images for a test sample and the corresponding ground truth image. (**b**) Distribution of average pixel error for test samples with a mean of 6.2% for 1000 test samples.


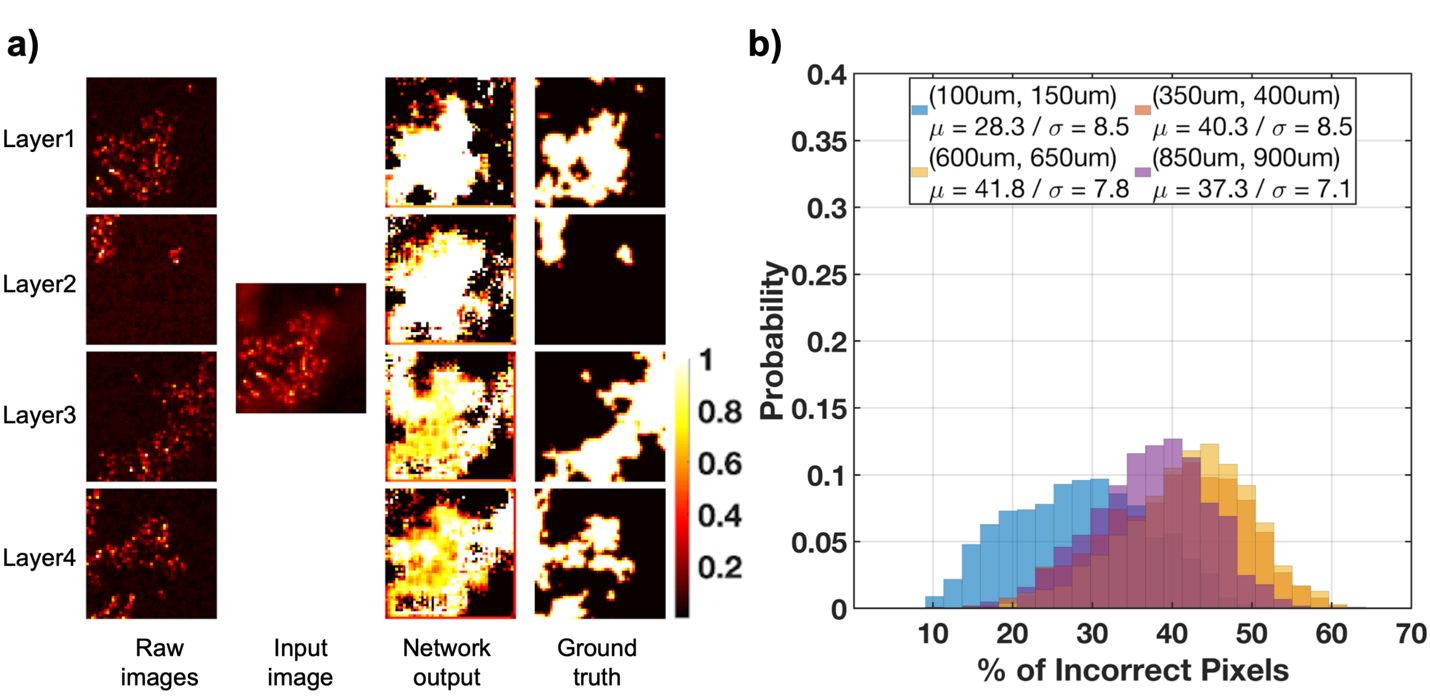


Figure S2. Test results of the CNN model with a single sensor: (**a**) Overlaid input image from 4 raw images corresponding to each layer, network output images and ground truth depth maps for each layer. (**b**) Distribution of average pixel error for test samples for each layer (starting from the closest one to the sensor) with averaged error rates of 28.3%, 40.3%, 41.8% and 37.3% for layers 1 to 4 respectively.


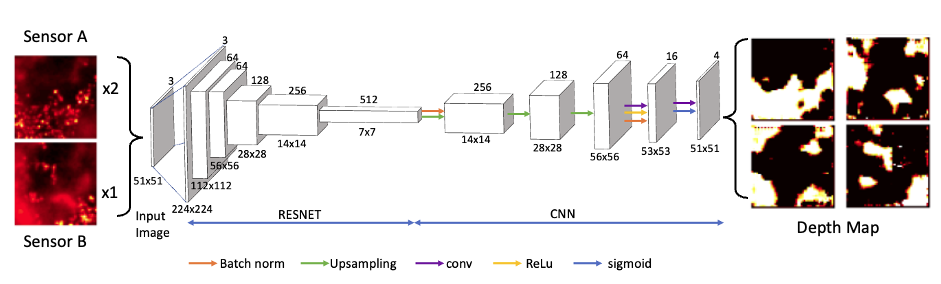


Figure S3. Architecture of the deep neural network model consisting of 18-layer ResNet and CNN with the corresponding input images from 2 sensors and the output depth maps for 4 layers. A replica of the image from sensor A is added to the 2 input images to comply with the 3-channel input of ResNet.

**
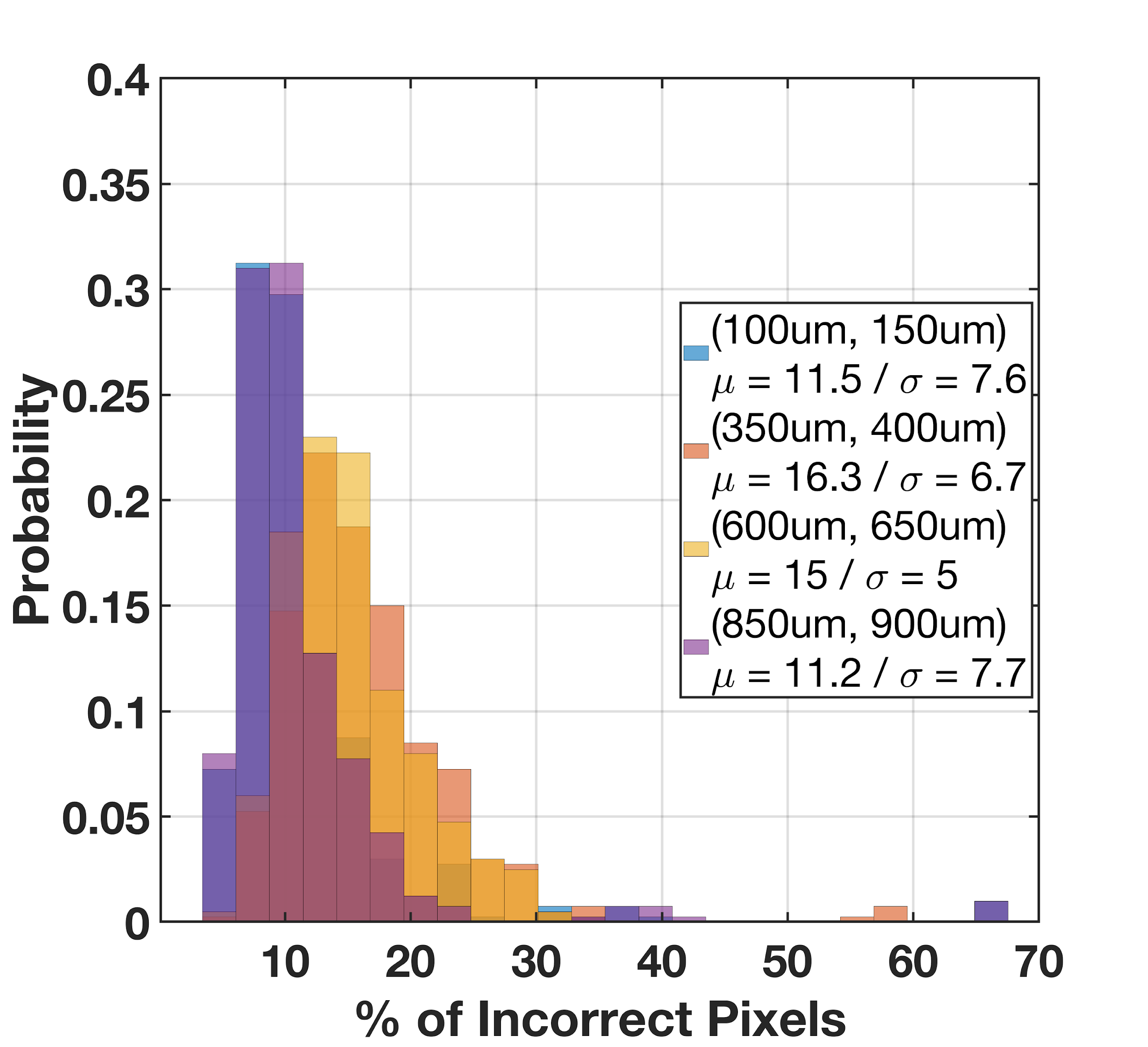
**

Figure S4. Performance of the ResNet+CNN model in identifying cell locations for each layer with average error rates of 11.5%, 16.3%, 15% and 11.2% for layers 1-4.
