# Supplementary material for: 3D Reconstruction of cellular images from microfabricated imagers using fully-adaptive deep neural networks
Source: Sci Rep. 2022 May 4;12:7229. doi: 10.1038/s41598-022-10886-6 (PMC9068918; doi:10.1038/s41598-022-10886-6)
Supplement: Supplementary file 2 — Supplementary Information 2. [file 41598_2022_10886_MOESM2_ESM.docx]

**Table S1.** Performance summary of 3 different trained depth estimation modules.

| **Architecture** | 6-layer CNN | 6-layer CNN | 18-layer ResNet + 2-layer CNN |
| --- | --- | --- | --- |
| **Application** | Depth estimation (3D reconstruction) | | |
| **No. of sensors** | 1 | 2 | 2 |
| **Layer 1 error rate (%)** | 28.3% | 12.2% | 11.5% |
| **Layer 2 error rate (%)** | 40.3% | 18.1% | 16.3% |
| **Layer 3 error rate (%)** | 41.8% | 18.4% | 15.0% |
| **Layer 4 error rate (%)** | 37.3% | 12.0% | 11.2% |
| **Avg. error rate** | 36.9% | 15.2% | 13.5% |
